# The body fades away: investigating the effects of transparency of an embodied virtual body on pain threshold and body ownership

**DOI:** 10.1038/srep13948

**Published:** 2015-09-29

**Authors:** Matteo Martini, Konstantina Kilteni, Antonella Maselli, Maria V. Sanchez-Vives

**Affiliations:** 1 Institut d’Investigacions Biomèdiques August Pi i Sunyer (IDIBAPS), Barcelona, Spain; 2 Event-Lab, Facultat de Psicologia, Universitat de Barcelona, Barcelona, Spain; 3 Institució Catalana Recerca i Estudis Avançats (ICREA), Barcelona, Spain; 4 Departamento de Psicología Básica, Universitat de Barcelona, Barcelona, Spain

## Abstract

The feeling of “ownership” over an external dummy/virtual body (or body part) has been proven to have both physiological and behavioural consequences. For instance, the vision of an “embodied” dummy or virtual body can modulate pain perception. However, the impact of partial or total invisibility of the body on physiology and behaviour has been hardly explored since it presents obvious difficulties in the real world. In this study we explored how body transparency affects both body ownership and pain threshold. By means of virtual reality, we presented healthy participants with a virtual co-located body with four different levels of transparency, while participants were tested for pain threshold by increasing ramps of heat stimulation. We found that the strength of the body ownership illusion decreases when the body gets more transparent. Nevertheless, in the conditions where the body was semi-transparent, higher levels of ownership over a see-through body resulted in an increased pain sensitivity. Virtual body ownership can be used for the development of pain management interventions. However, we demonstrate that providing invisibility of the body does not increase pain threshold. Therefore, body transparency is not a good strategy to decrease pain in clinical contexts, yet this remains to be tested.

Visual feedback has been shown to effectively modulate pain sensations in experimental studies with both healthy participants and chronic pain patients^1^,^2^,^3^,^4^. In particular, there is increasing evidence from pain studies showing how vision of the body may lead to effective pain relief 5,6,7,8,9,10. For instance, in a seminal study by Longo and co-workers, it was shown that the vision of one’s hand produces analgesic effects compared to when the gaze is oriented toward an object or another’s hand^5^. In the same study the authors demonstrated that the attenuated behavioural response was paralleled by a significant decrease in the brain response elicited by the nociceptive stimuli. Further experimental research has confirmed that there is an effective interaction between the vision of the body and the sensation of pain. For example Mancini and co-workers demonstrated that the visual size of someone’s own hand manipulated through mirrors leads to modulation of the pain threshold. In particular these authors reported that the vision of a smaller hand seen through a concave mirror leads to a higher pain threshold, while the vision of a bigger hand through a convex mirror decreases pain perception^11^. The rubber hand illusion provided researcher with a paradigm to experimentally induce illusory ownership of external body parts, so that the fake limb is felt as belonging to one’s body^12^. However, there are contradictory findings regarding whether looking at an owned rubber hand is analgesic or not. Recently, it has been reported that the analgesic effect of looking at one’s own hand is not present when looking at an owned rubber hand^13^. However, a subsequent study using a similar paradigm found the opposite results, with an effect comparable to that obtained by the vision of the real limb^7^. Moreover, further experimental evidence has shown how the analgesic effect of looking at one’s own body is present also when the painfully-stimulated volunteer is looking at an avatar’s limb, provided that this is felt as her/his own^8^. Furthermore, the vision of dynamic colour changes applied to the skin of the owned avatar’s limb modulates pain threshold with heat stimuli such that a reddish skin colour leads to lower threshold compared to the vision of a bluish one^1^. Hansel and co-workers reported that during an out-of-body experience, an increase in the self-identification with an avatar’s body seen in front of the participant is associated with an increase in pressure pain threshold^14^. Lately, following a similar paradigm, Romano and colleagues reported a reduced skin conductance response to painful stimuli when participants saw and identified themselves with the avatar body displayed in front of them^15^. All of these studies provide interesting insights on how the vision of the body can play a major role in the modulation of pain perception.

Body-related visual feedback is known to produce analgesic or pain relief effects in clinical populations as well. The experience of a phantom limb, typically reported by amputees, is often accompanied by painful sensations that are elicited or exacerbated by different physical and psychological factors^16^. Phantom limb pain has been treated by means of mirror therapy, where the patient can see an intact limb at the location where the phantom limb is felt to be, thus providing an illusory visual feedback that the lost limb is still there^3^. Also, it has been shown that viewing one’s painful limb as becoming smaller decreases the pain related to that limb^17^. The same manipulation though has been shown to lead to the opposite effect in healthy participants^11^, probably due to the different clinical conditions.

While the effects related to the vision of the body are classically related to dichotomous body *versus* no-body paradigms, namely to seeing or not seeing the body part, it remains unknown whether and how different levels of visibility of the body could affect pain perception. One could hypothesize that the vision of a body that fades away could increase analgesia by perceiving one’s own body as less likely to be harmed by the painful stimulus because it is fading away, or, conversely decrease analgesia due to the analgesic effect of the vision of the body^5^,^7^,^8^. Therefore an investigation on the effects of body transparency is needed. However, from a technical point of view, rendering a visible object “transparent” or “semi-transparent” is not trivial. For instance, paradigms relying on visual masking^18^ or on binocular rivalry^19^ provide only transient illusions and do not really offer valid solutions to make solid, non-transparent matter invisible. Also, these techniques usually use simple visual cues rather than complex physical entities such as human bodies. Immersive Virtual Reality (IVR) technology represents an effective tool as it allows the creation of sensory environments that can be replicated almost identically and that are under the full control of the experimenter^20^. Furthermore, not only does IVR allows one to feel immersed and present in a computer-generated environment, but it also makes possible the induction of the illusion of owning a virtual body^20^,^21^, a body that can be rendered with the morphological characteristics that the experimenter determines^22^. This illusion has been shown to affect the perceptual responses of participants^23^. So, thanks to its unique advantage to manipulate visual information in a controlled and systematic manner, IVR represents an excellent tool for investigating body-related human perception, including pain perception, under both healthy and clinical conditions.

Here we aimed at investigating whether and how much the illusion of body ownership over a virtual body and pain threshold change as the body becomes increasingly more transparent. We presented healthy participants with a co-located virtual body with four different levels of transparency, while participants were tested for pain threshold by increasing ramps of heat stimulation.

## Results

**Figure 1:**
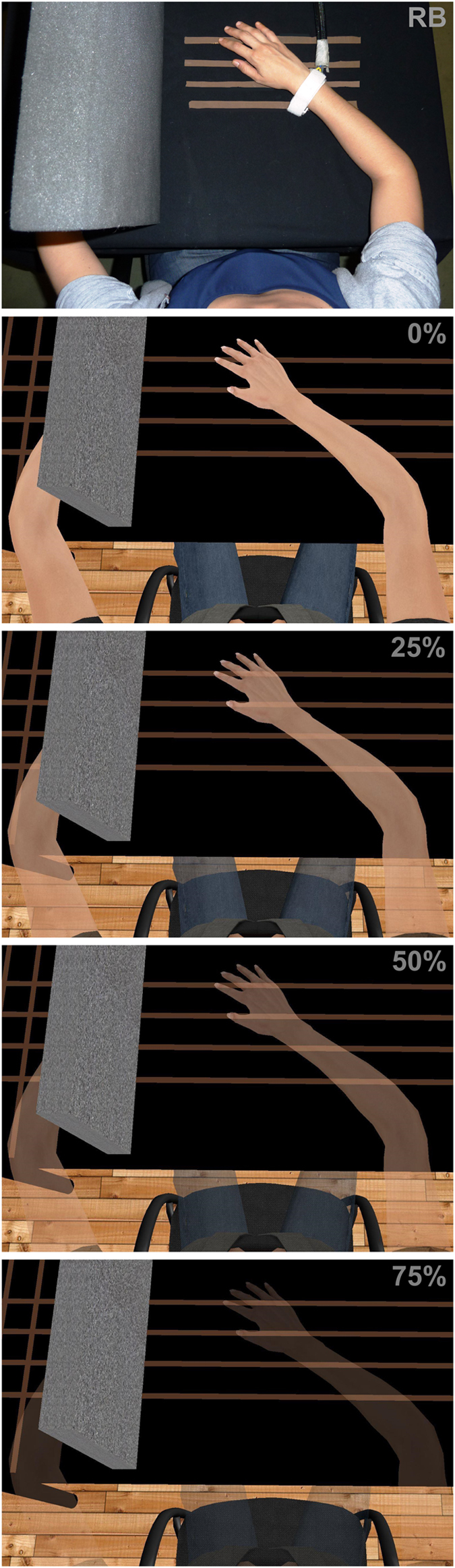
The experimental set-up (i.e. real body, RB) and the experimental conditions: From top to bottom, the avatar’s body is rendered fully opaque (0% transparency), or with 25%, 50% and 75% of transparency. The exemplifying picture was taken by MM and the person depicted is KK (both authors of the present manuscript). The virtual environment was programmed using the XVR system^38^ (VRMedia S.r.l., Pontedera, Italy) and the virtual body using the HALCA library^39^.

The experiment comprised different visual conditions where participants could see the avatar’s body from a first person perspective with different degrees of transparency (see Fig. 1). In particular, this within group design entailed a single factor, “transparency”, with 4 different levels (0%, 25%, 50%, 75% of transparency). The order of the conditions was counterbalanced between participants. Three heat ramps were provided for each condition, where participants were instructed to stop the stimulation as soon as they felt the heat to be painful. After each heat stimulation participants were also asked to rate the level of the illusion of ownership over the virtual limb (for further details see Methods section).

### Dependency of ownership on opacity/transparency of the virtual arm

**Figure 2:**
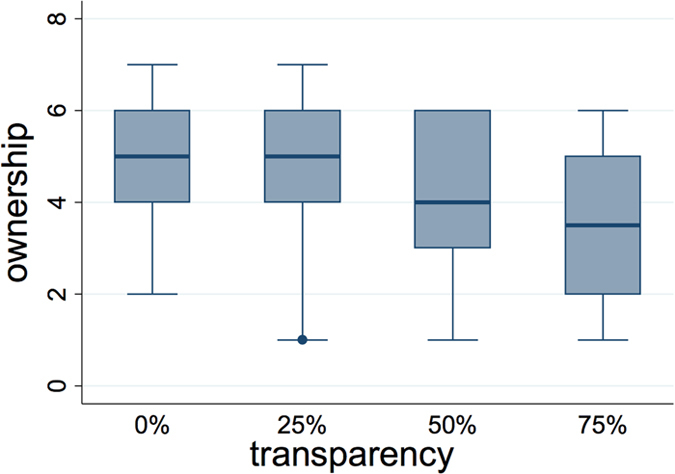
The box-and-whisker plot shows levels of ownership of the avatar’s body according to the different levels of body transparency (from 0% = fully opaque to 75% of transparency).

The reported levels of ownership showed a clear dependence on the transparency levels, being ownership lower with higher transparency (Fig. 2). Indeed we found a significant negative correlation (Spearman correlation: r_s_ = −0.32, p = 0.0017) between the two. Thus, in these conditions, the higher the level of transparency the lower the sense of ownership. An ordered logistic regression revealed that the factor “transparency” (treated as a numeric variable) was a significant predictor of “ownership” (treated as an ordered categorical variable), which was fit with a negative coefficient (c = −0.52) at a confidence level p = 0.0016. A Friedman One-Way Anova was then run to check whether there were different body ownership levels among the four conditions. This test showed that indeed the body ownership levels were significantly different under the diverse transparency conditions (χ^2^ = 15.87; p = 0.001). Single comparisons were run with matched pairs Wilcoxon tests. Together with the p-value we report the effect size in terms of the probability of superiority of dependent measures (PS_dep_): in the most transparent condition (75%) ownership was significantly lower than in the 50% (z = −2.57, p = 0.01, PS_dep_ = 0.79), 25% (z = −2.97, p = 0.003, PS_dep_ = 0.79) and 0% (z = −3.1, p = 0.002, PS_dep_ = 0.82) transparency conditions (see method sections for more details on how different levels of transparency were implemented); in the 50% condition ownership was significantly lower than in the 0% condition (z = −1.97, p = 0.048, PS_dep_ = 0.68); the rest of comparisons resulted in no significant difference (p = 0.13 for 25% vs 0% and p = 0.16 for 50% vs 25%), showing nevertheless a trend for lower ownership scores in the higher transparency condition.

### Dependency of pain threshold on opacity/transparency of the virtual arm

The data from our experiment show no direct dependence of the pain threshold (PT) on the transparency of the virtual body. In fact, a repeated measure one-way ANOVA with factor “transparency” gave no significant results (F(3,23) = 0.87, p = 0.46). Analogous results were found when including the temporal order of conditions as a covariate (ANCOVA), which would account for habituation effects (*transparency*: F(3,23) = 1.59, p = 0.2; *block*: F(3,23) = 57.9, p < 0.0001). The latter were nevertheless taken into account in the experimental design via the perfect balance in the order presentation of the four experimental conditions.

**Figure 3:**
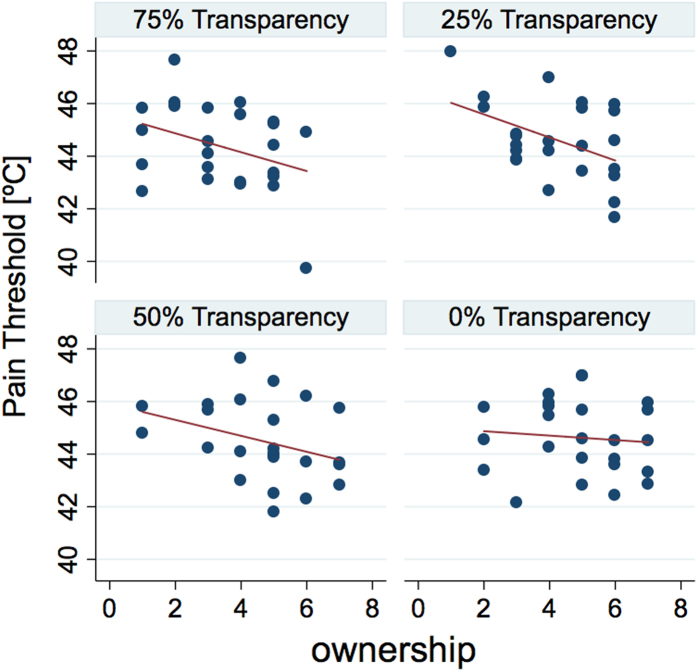
Scatterplots of ownership levels and pain threshold per participant and condition. Trend-lines show a trend or significant negative correlations only for the semi-transparent bodies (25%, 50% and 75%).

Interestingly, the data showed a negative correlation of PT with the level of ownership (Spearman correlation: r_s_ = −0.27, p = 0.007); note that higher PT values indicate a higher tolerance to painful stimuli, so this result suggests that the higher the sense of ownership the less the tolerance to painful stimuli. However, including all conditions together such correlation could be plagued by the autocorrelation bias. So, we ran the correlation separately for each level of transparency, and we found: [r_s_ = −0.30, p = 0.152] for 75% transparency; [r_s_ = −0.39, p = 0.064] for 50% transparency; [r_s_ = −0.42, p = 0.04] for 25% transparency; [r_s_ = −0.1, p = 0.46] for 0% transparency. Therefore, we found that the negative correlation between PT and ownership holds (i.e. is significant or in trend) only for the conditions with a transparent body (75%, 50%, 25%). No negative correlation is found for the case of a normal, fully opaque, virtual body (Fig. 3). Putting together data from the three conditions with a transparent body we found a significant negative correlation with PT [r_s_ = −0.33, p = 0.005], meaning that the higher the ownership of the transparent virtual arm, the lower the PT.

Not taking into account the level of ownership, PT shows no relationship with transparency (Spearman correlation: r_s_ = −0.03, p = 0.74).

## Discussion

Motivated by gathering new insights into the effects of visual exposure to the body and pain perception, and in an attempt to find a novel visual mechanism capable of modulating pain, in the present experiment we changed the virtual body visibility by making the avatar’s body progressively transparent. To our knowledge, this is the first study that addresses the influence of body visibility on both the sense of ownership and its impact on pain perception.

The results obtained from our set-up suggest that while the illusion of ownership is significantly reduced by increasing the transparency of the virtual body, pain threshold does not seem to be directly modulated by it. On the other hand, significant negative correlations were found between pain threshold and the subjective level of ownership of the transparent body. More specifically, this relation was found to hold for all the conditions in which participants were in view of a transparent body, but not when staring at a fully opaque virtual body. In other words, experiencing a greater sense of ownership over a transparent body results in a diminished pain threshold, and so in a higher sensitivity to painful stimuli. Importantly, our results are compatible with previous studies showing that the vision of one’s own body part (either real, dummy, or virtual) leads to analgesic effects (respectively^5^,^7^,^8^). Indeed there is no negative relationship between body ownership and pain threshold when the displayed body is normally displayed (0% transparency).

There are various possible interpretations for the negative relationship between pain and body transparency. First, it can be argued that a transparent body can imply some sort of weakness or vulnerability and therefore it can be perceived as more sensitive and susceptible to be hurt. In agreement with this argument, a recent study demonstrated that the illusion of owning a marble hand, induced by replacing the auditory impact of a hit on the participants’ hand with the sound of a hammer against a piece of marble, people perceived their hand to be harder and less sensitive^24^. Further, Osumi and coworkers recently reported that when participants are painfully stimulated while they experience the illusion of owning a rubber hand that is injured (i.e. presenting a bloody scar along the rubber limb), their pain threshold decreases^25^.

In addition, owning a transparent body may lead to general negative feelings toward the body and consequently, this attitude may influence the pain perception negatively. In agreement with this explanation, a recent study showed how unpleasant emotions toward a modified (specifically: magnified) image of one’s hand are related to lower pain thresholds^26^. Interestingly, in this study, no association with a lower pain threshold was found when the limb was not visually modified, which is in accordance with our findings. With an alternative interpretation we speculate that transparency may result in blurring the body limits, thus decreasing the predictability for potential injuries; hence, being uncertain about the body boundaries, the brain proceeds in a general lowering of the pain threshold as an alert system, strengthening the protective mechanisms.

Our results could also be complementary to those showing that seeing one’s own body produces analgesia and that this effect vanishes when the visual cue is something else (namely another’s body or an object)^5^,^8^. The neural underpinnings of this effect are associated to reduced activations of key areas of the putative ‘pain matrix’ during the vision of the body^27^. Indeed, studies with chronic pain patients and healthy participants point at the intracortical excitability in the primary somatosensory cortex as the mechanism underlying pain modulation^27^. The vision of the body would not only increase the intracortical inhibition within somatosensory cortex but it would also facilitate a reorganization of its somatosensory maps sharpening the receptive fields of the neurones^28^. These two mechanisms have been proposed to underlie the analgesia that follows the vision of the body, an effect that vanishes in the absence of such visual cue. In line with these proposed mechanisms, we found that there is a relationship between owning a virtual body that gets transparent and the loss of the analgesic effect derived from seeing one’s own body^5^.

In addition, our results reveal that the ownership of a virtual arm by means of co-location decays with the transparency of the arm; in other words, ownership of an opaque arm is significantly higher than that of a semi-transparent arm. This result is in agreement with several findings in the literature on body ownership illusions that emphasize the role of top-down influences in modulating the level of illusion, in terms for example of body volume^29^, body length^30^, or body connectivity^31^ (for a review see^32^). Our results of reduced body ownership scores toward a semi-transparent arm may apparently disagree with the results by Guterstam and coworkers who recently demonstrated that people can experience strong sensations of body ownership toward an empty area of the space when congruent visuotactile correlations are provided to the participant’s limb and the empty area^33^. However, any comparison between the two studies should be done with caution due to significant methodological differences; for example, in Guterstam’s study, there was no body part displayed, while in our experiment a body was always shown under different transparency levels. In addition, these authors provided visuotactile stimulation in order to induce the “ownership” of the empty area, while in our study the first person perspective of a collocated avatar’s body was sufficient to achieve the virtual body illusion^34^,^35^. We did not provide additional visuotactile stimulation in order to avoid ceiling effects on the level of the ownership illusion, which might have hindered the detection of modulations effects associated to different levels of transparency.

## Conclusion

A number of studies have been conducted to explore how bodily illusions may affect our perception. However, how body visibility affects body ownership and pain processing is new since it is an effect hard to achieve in the real world. Such approach not only expands our knowledge about how we perceive our body and interact with it and the environment, but it can also be a critical tool for the development of pain management interventions relevant for clinical therapy^36^. In the present study we found that the vision of a transparent body may lead to lower levels of ownership over that body. Participants reporting stronger ownership over the transparent body showed lower levels of pain threshold. Therefore, although visual transformations of the owned virtual body can lead to useful applications in therapeutical settings, making the body transparent does not seem to be useful to increase the pain threshold. From these observations we could predict that if a patient reports pain in a limb, making this limb (virtually) transparent would not decrease his/her pain. However, such hypothesis remains to be confirmed by future studies. Here we studied pain threshold on healthy subjects, however chronic pain has additional emotional and psychological components, and therefore the transparency of the painful limb could have a different impact in this clinical population.

## Methods

### Participants

24 right-handed female healthy participants (age: 21.1 ± 1.8) were recruited for the experiment. We intentionally chose participants of the same gender given that men and women adapt differently (sensory habituation) to pain stimuli^37^. All participants had normal or corrected-to-normal vision and no history of neurological or psychological disorders. Also, any condition potentially interfering with pain sensitivity (e.g. drug intake) was considered as a further exclusion criterion. Upon arrival at the laboratory they were asked to read and sign a consent form and the experiment was carried out in accordance with the regulations of the local ethics committee (Comité Ético de Investigación Clínica de la Corporación Sanitaria Hospital Clínic de Barcelona) and with the declaration of Helsinki. The study was approved by the ethics committee of the Hospital Clinic of Barcelona. All participants received a monetary compensation for their participation (10 €).

### Virtual reality system

The stereoscopic head-mounted display (HMD) was a NVIS SX111 with a resolution of 1280 × 1024 per eye and a total field of view of 102° × 64°, displayed at 60Hz. The head-tracking was realized with a 6-DOF Intersense IS-900 device (InterSense, Billerica, USA). The virtual environment was implemented using the XVR system^38^ (VRMedia S.r.l., Pontedera, Italy) and the virtual body was displayed using the HALCA library^39^. Transparency of the virtual body was achieved by adjusting the value of alpha channel of the texture of the virtual body to levels of either 100% (maximum opacity), 75%, 50% or 25% (very transparent) (see Fig. 1). Noise isolation was ensured by the administration of pink noise through a surround audio system (Creative technology Ltd., Singapore), with a constant volume set at 65 dB.

### Pain threshold measurement

Thermal heat stimuli were delivered by means of a Somedic-Thermotest machine (Somedic, Stockholm, Sweden) with a 2.5 cm × 5.0 cm thermode tied with a Velcro strap on the front of the participant’s right forearm, close to the distal extreme of the radius (see Fig. 1). Pain thresholds were assessed with the method of limits^40^. The probe temperature was increased from normal skin temperature (constant baseline temperature = 31 °C) at 2 °C/s. The temperature stimulation was provided in a continuous fashion, gradually and constantly increasing from the baseline temperature till the subjective pain threshold level. Participants were instructed to press a button with their left hand as soon as they perceived the stimulation on their right hand as being painful. Immediately after pushing the kill-switch button, the probe temperature rapidly decreased to the baseline temperature. For safety reasons, maximal temperature was set at 48 °C.

### Procedure

Participants sat comfortably on a chair with both arms resting on a table covered with a black cloth as shown in Fig. 1. Before starting the main part of the experiment, participants were given 2-3 heat stimuli to familiarize them with the heat ramps. Afterwards, the participant put on the HMD, the room’s lights were turned off and the pink noise played. The posture of the participant was calibrated in order to ensure a close co-location of the real body with the virtual one. The HMD allowed participants to experience an immersive virtual environment around them and to see a virtual body from a first-person perspective so that, when they looked down towards their own body, they could see the virtual body in place of their own. Participants were asked to keep still throughout the experiment. Importantly, no other sensory correlations were provided (visuo-tactile or visuo-motor correspondences) since a first person perspective over a realistic fake humanoid body has been shown to be sufficient in eliciting a strong body ownership illusion^34^. Four brown stripes of tape were stuck on the real table and displayed in the virtual table to facilitate the perception of a transparency of the avatar’s arm and to boost the visuo-tactile correspondence between the real and the virtual hand (both the real and the virtual hand touching the first stripe with the finger and the second stripe with the thumb).

Before each condition, participants were given approximately one minute to familiarize with the virtual room in which they were in and with the virtual body. The experiment consisted of four different conditions, presented in blocks, with the avatar’s body transparency set at 0%, 25%, 50% or 75%. All participants completed the four conditions, with the order of the blocks balanced across participants. Each condition started with the participant looking at and describing the entire body and then focussing on the avatar’s right wrist (i.e. where the painful stimuli came from). The inter-stimulus interval was set at around 40 seconds and three heat ramps were provided for each condition.

Within each trial, about 2 seconds after the thermal stimulation had been stopped, a “beep” sound prompted the participants to judge the level of ownership over the virtual limb. They were instructed to verbally report a number on a scale from 1 to 7 (“1” = not at all, “7” completely) to reply to the question: “Did you feel as if the virtual right arm was your own right arm?”. Participant’s ratings were promptly annotated by the experimenter.

## Additional Information

**How to cite this article**: Martini, M. *et al.* The body fades away: investigating the effects of transparency of an embodied virtual body on pain threshold and body ownership. *Sci. Rep.* **5**, 13948; doi: 10.1038/srep13948 (2015).
